# Research trends in the relationship between vitamin D and type 2 diabetes mellitus: a 20-year bibliometric and visualization analysis

**DOI:** 10.3389/fendo.2024.1421953

**Published:** 2024-08-13

**Authors:** Ruijun Xu, Xuejing Shao, Huibo Qiao, Han Yan, Yi Xue

**Affiliations:** ^1^ Department of Endocrinology, Affiliated Wujin Hospital of Jiangsu University, Changzhou, Jiangsu, China; ^2^ Department of Endocrinology, Wujin Clinical College of Xuzhou Medical University, Changzhou, Jiangsu, China

**Keywords:** vitamin D, type 2 diabetes, bibliometrics, Citespace, VOSviewer

## Abstract

**Introduction:**

Vitamin D has a significant correlation with type 2 diabetes. Insufficient levels of vitamin D can cause insulin resistance, which impairs the ability of cells to respond to insulin and worsens the progression of diseases. Furthermore, vitamin D has the potential to enhance the release of insulin, enhance the regulation of blood sugar levels, and reduce the glycemic index. Research has indicated that insufficient levels of vitamin D may elevate the likelihood of experiencing complications related to type 2 diabetes, including cardiovascular disease and neuropathy. This study employed bibliometric analysis to investigate recent advancements in research about the relationship between vitamin D and type 2 diabetes.

**Methods:**

We searched for articles on the topic of vitamin D and type 2 diabetes published between January 1, 2004, and December 31, 2023. The search was performed on February 20, 2024, using the Web of Science Core Collection (WoSCC). Utilizing VOSviewer and CiteSpace, we conducted bibliometric analysis and visualization.

**Results:**

A comprehensive study was conducted on a total of 1362 papers pertaining to the relationship between vitamin D and type 2 diabetes. The United States had the biggest number of publications and the highest effect among these articles. Within the top 10 most published journals, the journal “DIABETES CARE” has the highest H-index, indicating its significant influence in this field of study. Currently, there is an extensive body of research on the supplementation of vitamin D for the improvement of type 2 diabetes and prevention of complications in type 2 diabetes, as well as its related mechanisms. Research related to bone turnover and peripheral neuropathy represents a promising area for future studies.

**Conclusion:**

Overall, bibliometrics may assist researchers in comprehending the trajectory, significant themes, and scholarly influence of the field concerning vitamin D and type 2 diabetes. This, in turn, offers substantial backing for future studies that delve further into the subject matter.

## 1 Introduction

Vitamin D is crucial in the development and progression of type 2 diabetes mellitus and its associated problems. Type 2 diabetes mellitus is a long-term metabolic disorder characterized by insulin resistance and inadequate insulin production, resulting in high levels of glucose in the blood (1). Insulin resistance is a significant pathological mechanism that occurs in the initial phases of type 2 diabetes. Vitamin D is linked to insulin receptors in both muscle cells and fat cells. Sufficient consumption of vitamin D enhances insulin sensitivity in these cells and decreases insulin resistance (2). Furthermore, vitamin D has the potential to improve insulin resistance by regulating inflammatory responses and cellular communication pathways (3). Vitamin D also regulates the release of insulin. Vitamin D receptors have a broad presence throughout the pancreas and are linked to the regulation of insulin release and the functioning of insulin-producing cells. Several studies indicate that sufficient consumption of vitamin D may enhance insulin secretion and enhance glycemic management (4). The glycemic index is an important factor in the dietary management of individuals with type 2 diabetes since it measures how quickly foods increase blood glucose levels. Consuming vitamin D may potentially decrease the glycemic index of foods, leading to a reduction in blood sugar fluctuations and promoting better regulation of blood sugar levels (5). Inadequate levels of vitamin D can result in heightened problems associated with type 2 diabetes. For instance, a lack of vitamin D may elevate the likelihood of experiencing consequences such as cardiovascular disease, kidney disease, and neuropathy (6, 7). In recent years, there has been significant scientific research conducted on the relationship between vitamin D and type 2 diabetes, yielding positive results. The present study highlights the importance of maintaining adequate levels of vitamin D for the prevention and treatment of type 2 diabetes, offering guidance for future research and therapeutic use.

Bibliometrics is the field that focuses on analyzing and evaluating many aspects of scientific literature, such as its quantity, quality, citation patterns, and distribution across different disciplines (8). Bibliometrics allows for the quantitative analysis of trends and developments in scientific research. It enables the assessment of the influence and quality of academic outcomes, as well as the identification of collaborative links and disciplinary intersections within the academic community. Bibliometrics encompasses a wide range of academic literature, including research papers, monographs, patents, and conference papers. Bibliometric methods allow for the creation of literature databases, examination of literature citations, evaluation of academic performance for both institutions and academics and the provision of an unbiased foundation for scientific research policy development and academic assessment. Bibliometrics is crucial in scientific research administration, academic evaluation, and the formulation of scientific research policies (9).

Although there has been a significant amount of literature on the relationship between vitamin D and type 2 diabetes in the past twenty years, no bibliometric study has been carried out on this topic. In light of this, we undertook an extensive bibliometric review of the literature pertaining to the relationship between vitamin D and type 2 diabetes, encompassing the years 2004 to 2023. Through the examination of the published articles, we can determine the countries, institutions, authors, and journals that have the greatest quantity of publications. By analyzing the relevant keywords, we may gain a deeper understanding of the current areas of focus in research and predict future trends. This analysis can assist researchers in selecting their research paths and identifying emerging areas of study.

## 2 Materials and methods

### 2.1 Data sources and retrieval strategies

**Figure 1:**
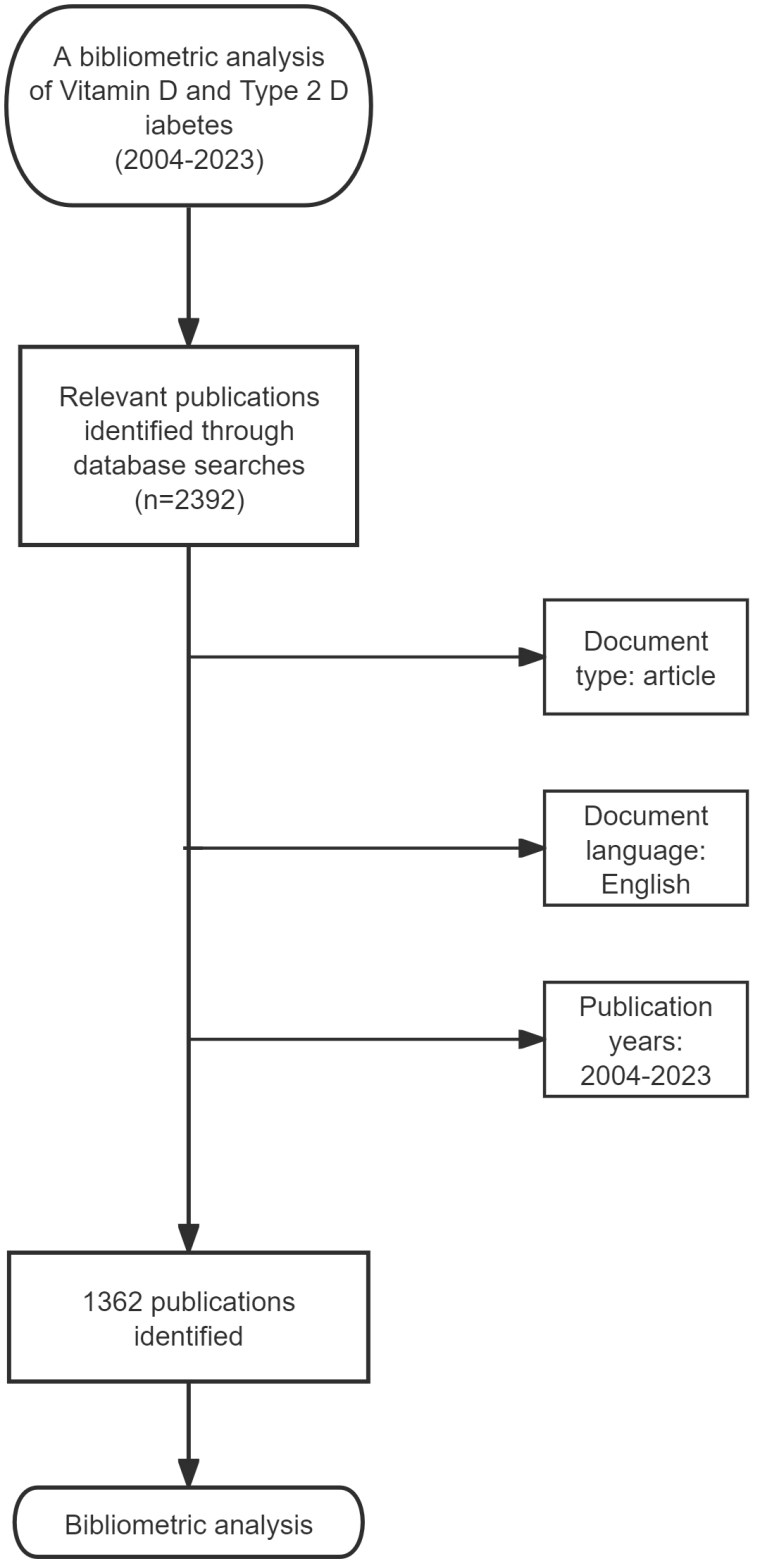
Flow chart of the study.

On February 20, 2024, we conducted a search on the Web of Science Core Collection (WoSCC) for items that were published between January 1, 2004, and December 31, 2023. To mitigate the impact of frequent database revisions, we successfully conducted the search and retrieval of the data within a single day. The search approach for obtaining papers in WoSCC is as outlined: The search terms include “TS = (Diabetes Mellitus Type 2)” OR “TS = (Type 2 Diabetes Mellitus)” OR “TS = (Type 2 Diabetes)” OR “TS = (Diabetes Mellitus Type)” OR “TS = (Type II Diabetes Mellitus)” OR “TS = (Type II Diabetes)” AND “TS = (Vitamin D)”. The search is limited to articles. To guarantee the precision of our bibliometric analysis, we conducted a comprehensive evaluation of each retrieved article by carefully evaluating its title, abstract, and year of publication. The study employed the following exclusion criteria: (1) articles that were not related to the topic of vitamin D and type 2 diabetes; (2) documents in formats other than articles, such as editorials, letters, reviews, and conference abstracts; (3) duplicate publications; and (4) publications that were not in the English language. Ultimately, the research resulted in the identification of 1,362 publications about the relationship between vitamin D and type 2 diabetes (**Figure 1**).

### 2.2 Bibliometric analysis

We further conducted a bibliometric analysis of 1,362 publications related to vitamin D and type 2 diabetes from January 1, 2004, to December 31, 2023. Based on the bibliometric analysis methods from previous studies, this study utilizes analysis software such as R version 4.3.2, VOSviewer, and CiteSpace (10–15). R software serves as a potent statistical programming tool, equipped with extensive capabilities for data handling and visualization (16). It boasts a plethora of extendable packages, such as bibliometrix and biblioshiny, which offer comprehensive functionalities for bibliometric analysis (17, 18). In this study, the bibliometrix package in R software was employed for initial publication statistics (17). VOSviewer specializes in bibliometric network visualization, particularly adept at generating scientific knowledge maps. This research leveraged VOSviewer to visualize co-authorship and time-based network analyses (11, 19). Additionally, ArcMap 10.8.1, known for geospatial analysis and cartographic visualization, was used to create a global map of research distribution (20). CiteSpace, known for analyzing citation networks and keyword co-occurrences, was utilized to identify citation burst years and develop a dual-map overlay (21).

## 3 Results

### 3.1 Overview

**Figure 2:**
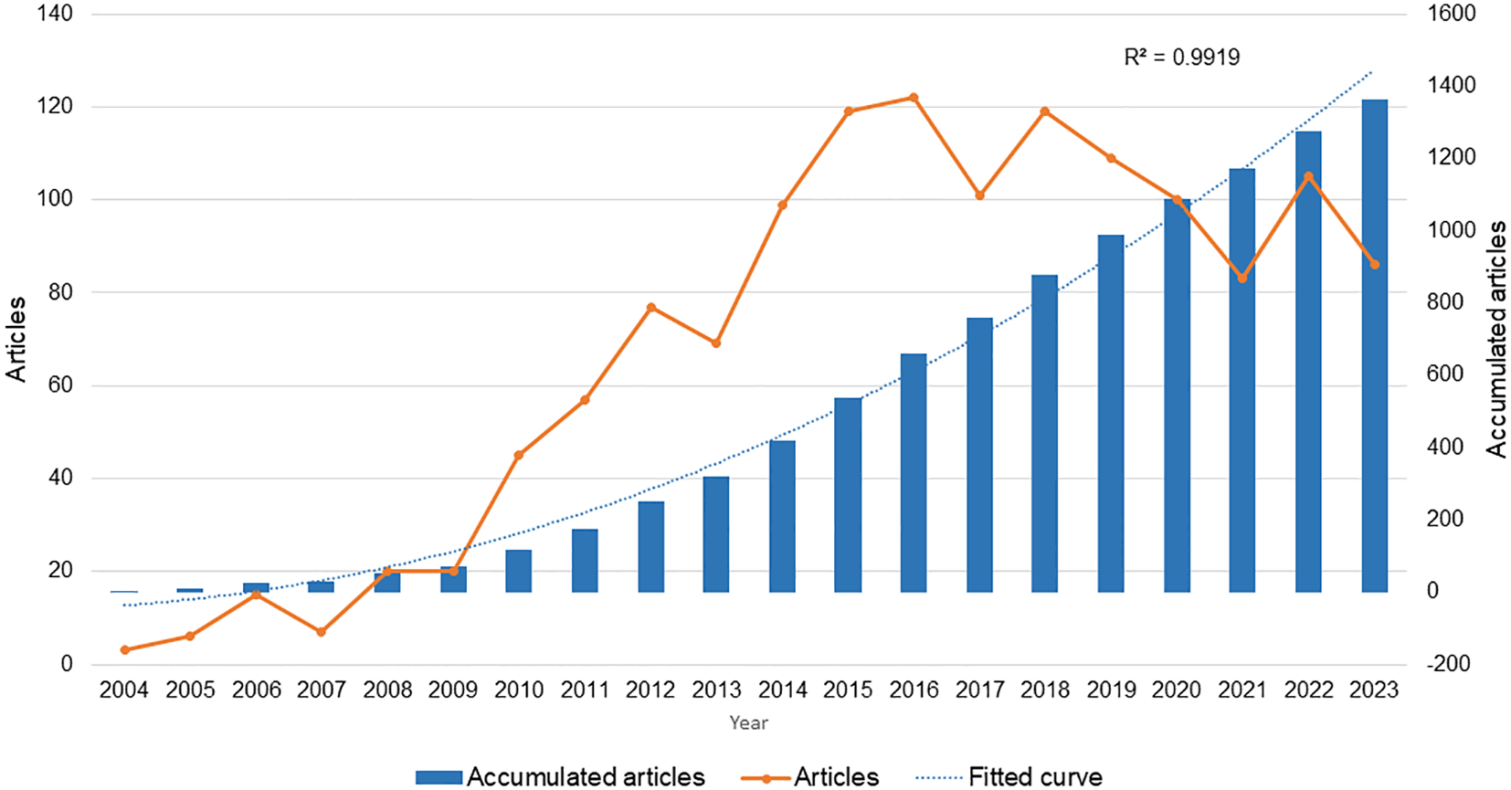
Number of publications per year and cumulative number of publications per year.

A total of 1362 articles were included in this study (**Figure 2**). Between 2004 and 2016, the number of articles had a predominantly rising trajectory. Nevertheless, between 2016 and 2023, there was a substantial decrease in the quantity of publications. The cumulative number of articles exhibited a consistent annual growth from 2004 to 2023. In 2020, the total number of articles published on the topic of vitamin D and type 2 diabetes exceeded one thousand.

### 3.2 Analysis of country and region publications

**Figure 3:**
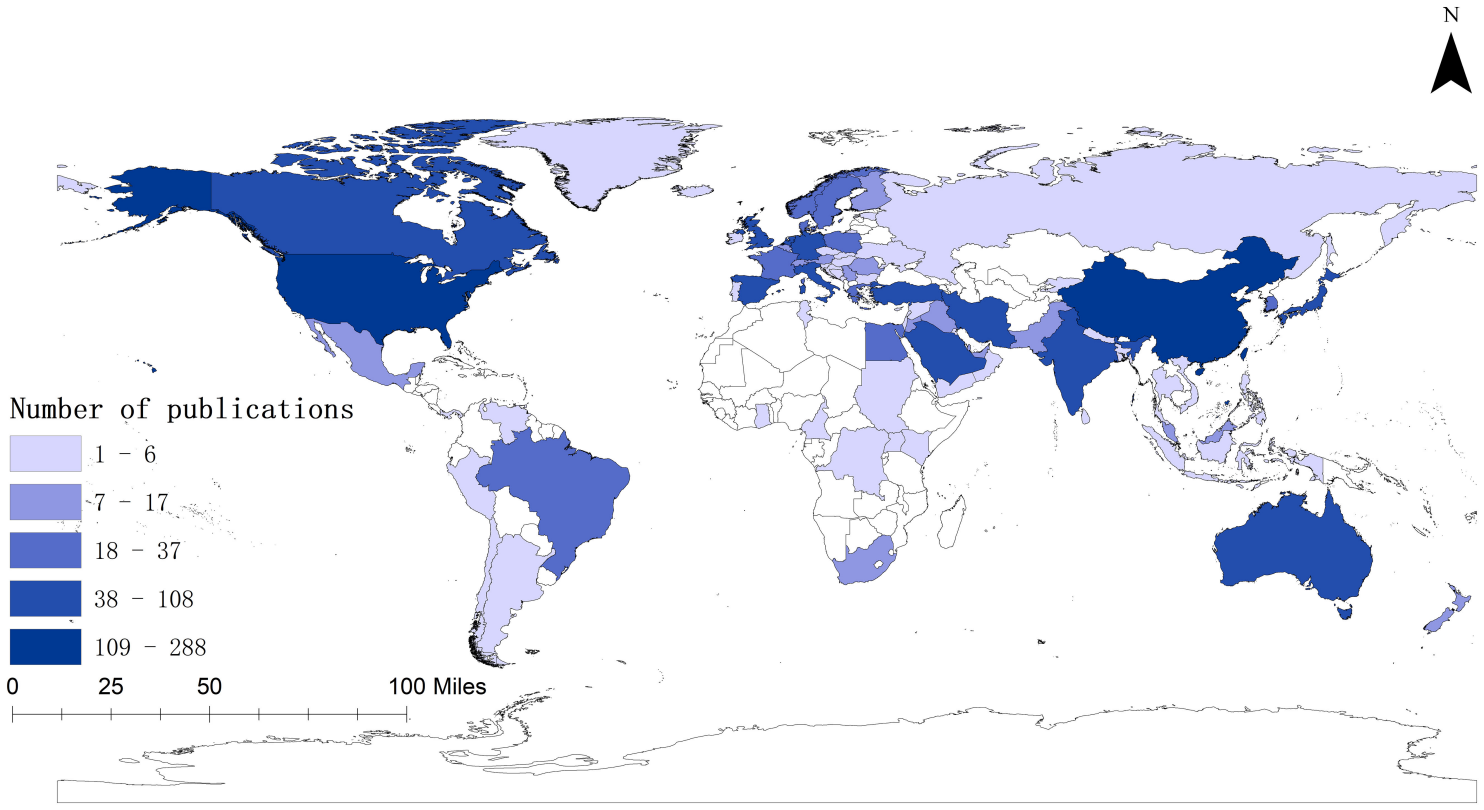
World map displaying the global distribution of research on Vitamin D and Type 2 Diabetes.

**Figure 3** indicates that there were 1362 research publications published on the topic of vitamin D and type 2 diabetes, spanning over 87 distinct nations. The majority of these articles originated from the United States and China, with notable contributions from the United Kingdom, Iran, and Italy (**Table 1**). Significantly, the United States achieved the top position in the total citation index, showcasing its supremacy in vitamin D and type 2 diabetes research.

**Table 1:** Top 10 countries with most publications.

Rank	Country	TLS	TP	TC
1	USA	181	288	16277
2	China	77	279	4943
3	United Kingdom	177	108	6516
4	Iran	16	83	2369
5	Italy	78	65	2586
6	Australia	55	54	2711
7	India	33	54	1004
8	Saudi Arabia	52	50	888
9	Germany	102	49	2647
10	Japan	7	49	1119

We performed bibliometric research to investigate the collaborative connections between countries and regions. The United States and China exhibited the most frequent instances of collaboration, surpassing the United States and the United Kingdom, which occurred 27 and 14 times, respectively. To better understand the extent of collaboration among these 87 countries, we undertook a co-authorship analysis.

**Figure 4:**
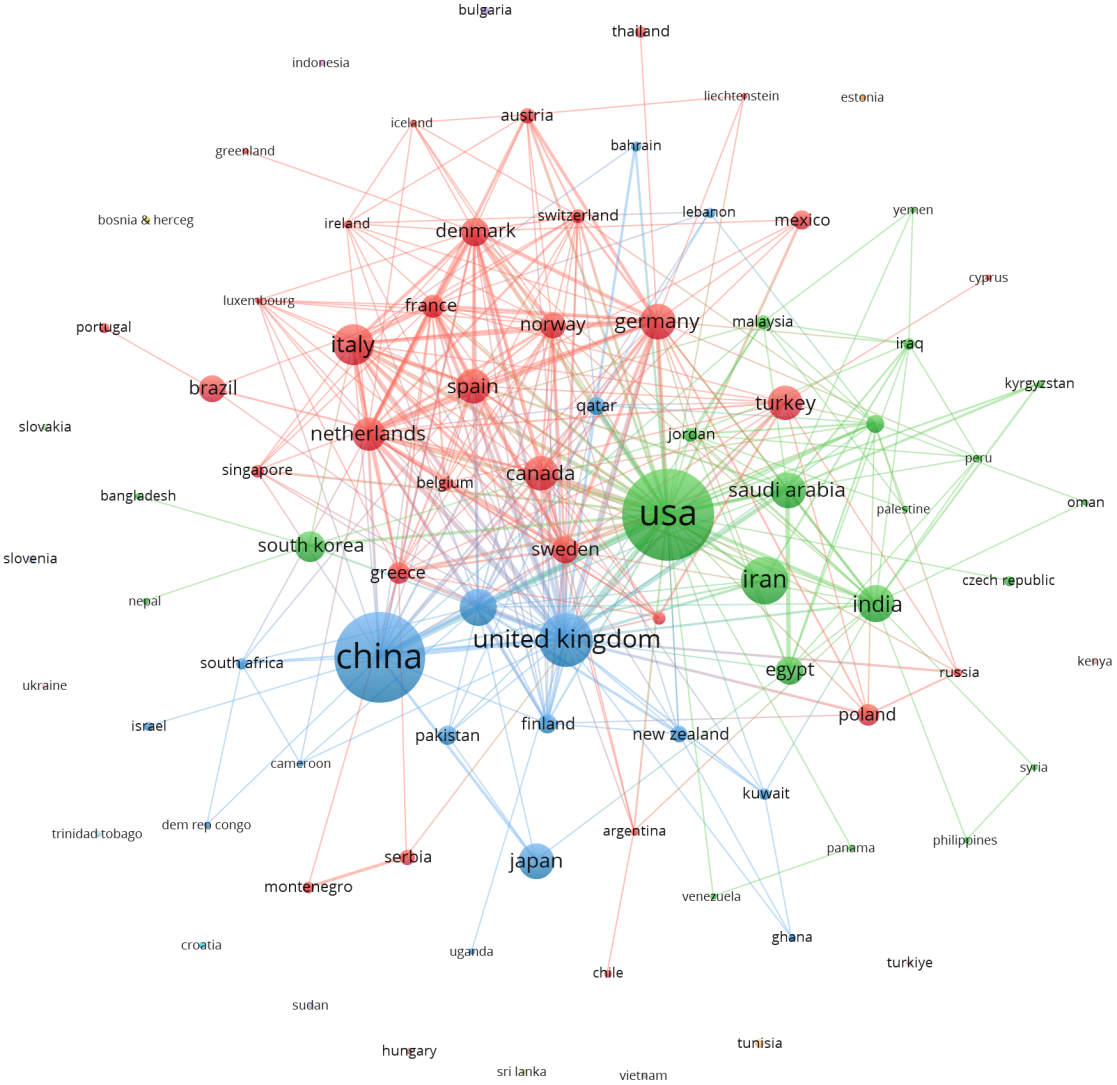
Network clustering of country co-authorship analysis.

The clustering network visually represents the number of publications based on the size of the circles (**Figure 4**). The hue of the circles corresponds to the degree of collaboration within the study team. The red cluster contains 32 items, the green cluster contains 22 elements, and the blue cluster contains 17 elements. Italy, Canada, and Brazil belong to the red cluster, while the United States, Iran, and India are part of the green region. China and the United Kingdom are grouped in the blue region. In the case of networks that have temporal overlap, the color of the circle represents the mean year of publication for each country within a specific study area. The analysis of the time-overlapping network suggests that China entered the field later than countries like the United States and the United Kingdom, who were the pioneers in this area (**Supplementary Attachment 1**).

### 3.3 Analysis of institution publications

There have been 2,120 academic institutes that have carried out and published research on the relationship between vitamin D and type 2 diabetes. Out of them, 174 institutions have published a minimum of five articles. **Table 2** provides the ranking of the top 10 colleges based on the number of articles they have published. Tehran University of Medical Sciences and Health Services has published the greatest quantity of publications, with a total of 33 papers. Harvard University and King Saud University have published 30 and 29 publications, respectively, following the top-ranked university.

**Table 2:** The top 10 institutions with the most publications.

Rank	Institution	TLS	TP	TC	Country
1	Tehran University of Medical Sciences and Health Services	28	33	738	Iran
2	Harvard University	67	30	3821	USA
3	King Saud University	23	29	643	Saudi Arabia
4	Shanghai Jiao Tong University	11	25	419	China
5	Tufts Medical Center	87	24	2147	USA
6	Brigham and Women’s Hospital	59	21	2691	USA
7	Tufts University	66	19	2221	USA
8	University of Toronto	45	18	978	Canada
9	Shahid Beheshti University of Medical Sciences	13	17	721	China
10	University of Copenhagen	41	16	813	Denmark

**Figure 5:**
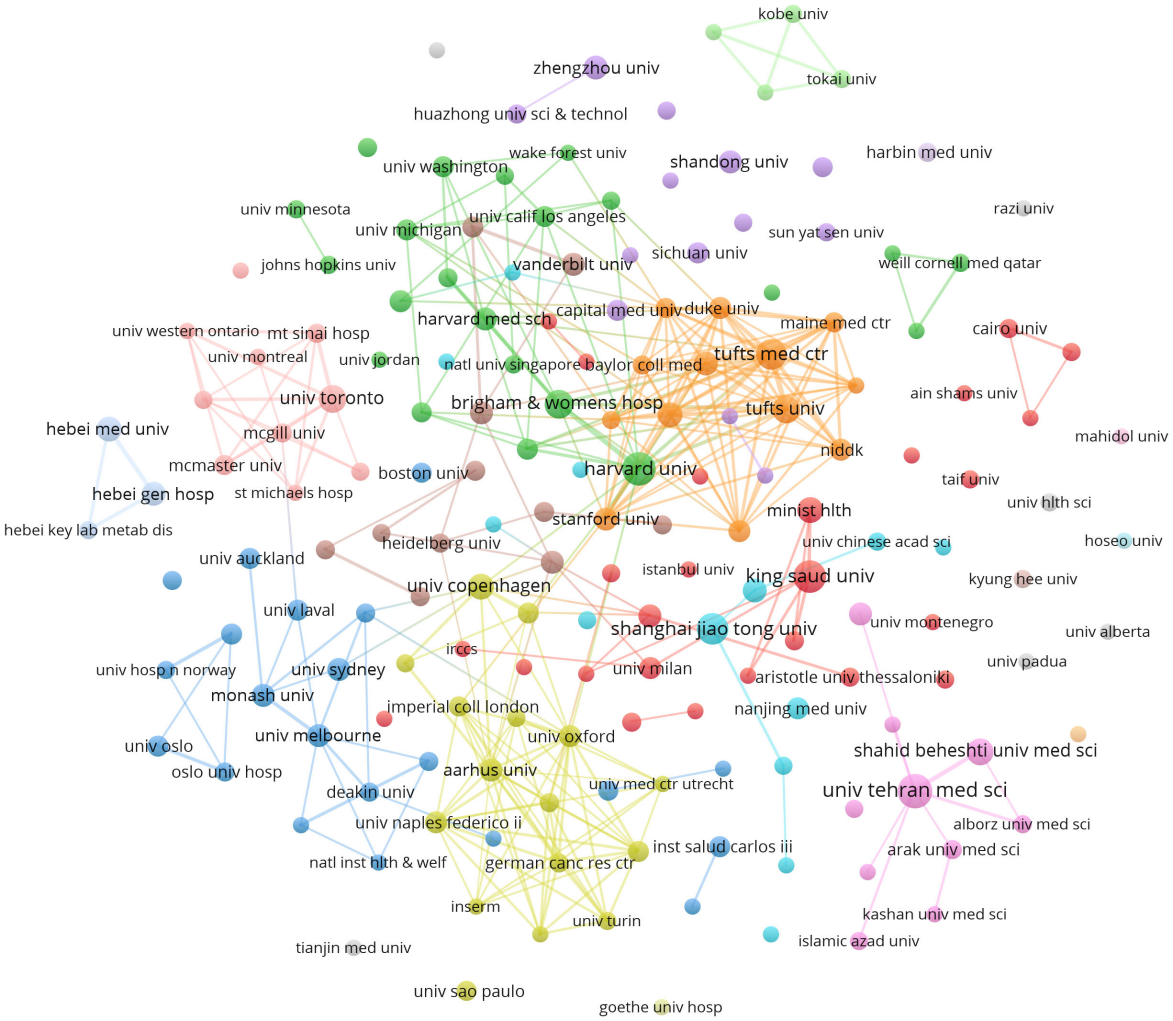
Network clustering of institution co-authorship analysis.

We conducted a cluster analysis on the 174 institutions, as shown in **Figure 5**. The red cluster, which primarily comprises 26 universities, predominantly from the United States, is the largest among all. Harvard University, along with other prominent U.S. research institutions, played a pivotal role in the initial advancement of vitamin D and its relationship to type 2 diabetes. After 2018, several Chinese research organizations increased their participation in studies on vitamin D and type 2 diabetes (**Supplementary Attachment 1**).

### 3.4 Analysis of journals

The study’s articles were disseminated among 469 distinct journals. **Table 3** displays the leading 10 publications together with their most recent impact factors for the year 2022. Five of the top 10 journals are in the highest quartile (Q1) of the JCR. H The three journals with the highest h-index are DIABETES CARE, JOURNAL OF CLINICAL ENDOCRINOLOGY & METABOLISM, and PLOS ONE.

**Table 3:** Top 10 most published journals.

Rank	Source	TP	TC	H-index	IF2022	JCR
1	NUTRIENTS	50	513	12	5.9	Q1
2	PLOS ONE	41	1400	20	3.7	Q2
3	DIABETES CARE	38	4439	34	16.2	Q1
4	JOURNAL OF CLINICAL ENDOCRINOLOGY & METABOLISM	33	2222	23	5.8	Q1
5	BMC ENDOCRINE DISORDERS	28	268	9	2.7	Q4
6	FRONTIERS IN ENDOCRINOLOGY	24	207	6	5.2	Q1
7	AMERICAN JOURNAL OF CLINICAL NUTRITION	23	340	19	7.1	Q1
8	SCIENTIFIC REPORTS	22	308	10	4.6	Q2
9	DIABETES METABOLIC SYNDROME AND OBESITY-TARGETS AND THERAPY	21	101	7	3.3	Q3
10	DIABETES RESEARCH AND CLINICAL PRACTICE	21	738	12	5.1	Q2

Dual-map overlay is a visualization technique that, by analyzing the citation relationships between disciplines, reveals the interactions and influences among different disciplines, aiding in the understanding of knowledge flow and integration between fields. In **Supplementary Attachment 1**, the journals on the left are the cited journals, whereas those on the right are the citing journals, with lines indicating the citation pathways. In this analysis, three key reference paths have been identified. The yellow path signifies that articles from journals in Molecular/Biology/Genetics are cited by articles in Molecular/Biology/Immunology. On the other hand, the green citation path indicates that articles from Molecular/Biology/Genetics and Medicine/Medical/Clinical journals are primarily cited by articles published in Health/Nursing/Medicine journals.

### 3.5 Most cited publications

The most cited publications may encompass seminal works, significant research findings, pivotal research breakthroughs, or innovative studies. Through the analysis of these extensively referenced articles, scholars can gain insight into the prevailing academic patterns, significant research pathways, and notable contributions made by exceptional researchers in the field (22). **Table 4** displays the top 10 articles that have received more than 300 citations.

**Table 4:** Top 10 most frequently cited documents.

Rank	Title	Year, Journal	First author	Total citations
1	Hypovitaminosis D is associated with insulin resistance and β cell dysfunction	2004, AMERICAN JOURNAL OF CLINICAL NUTRITION	Boucher, Barbara J	1305
2	Vitamin D and calcium intake in relation to type 2 diabetes in women	2006, DIABETES CARE	Pittas, Anastassios G.	562
3	Selective vitamin D receptor activation with paricalcitol for reduction of albuminuria in patients with type 2 diabetes (VITAL study): a randomised controlled trial	2010, LANCET	de Zeeuw, Dick	547
4	Vitamin D supplementation reduces insulin resistance in South Asian women living in New Zealand who are insulin resistant and vitamin D deficient - a randomised, placebo-controlled trial	2010, BRITISH JOURNAL OF NUTRITION	von Hurst, Pamela R.	477
5	Baseline serum 25-hydroxy vitamin D is predictive of future glycemic status and insulin resistance - The Medical Research Council Ely Prospective Study 1990-2000	2008, DIABETES	Forouhi, Nita G.	464
6	Vitamin D improves endothelial function in patients with Type 2 diabetes mellitus and low vitamin D levels	2008, DIABETIC MEDICINE	Sugden, Jacqui A.	444
7	Blood 25-Hydroxy Vitamin D Levels and Incident Type 2 Diabetes	2013, DIABETES CARE	Song, Yiqing	371
8	Vitamin D Supplementation and Prevention of Type 2 Diabetes	2019, NEW ENGLAND JOURNAL OF MEDICINE	Pittas, Anastassios G.	352
9	Risk factors for type 2 diabetes mellitus: An exposure-wide umbrella review of meta-analyses	2018, PLOS ONE	Bellou, Vanesa	340
10	Effects of vitamin D and calcium supplementation on pancreatic β cell function, insulin sensitivity, and glycemia in adults at high risk of diabetes: the Calcium and Vitamin D for Diabetes Mellitus (CaDDM) randomized controlled trial	2011, AMERICAN JOURNAL OF CLINICAL NUTRITION	Mitri, Joanna	314

### 3.6 Analysis of the author

This study conducted a thorough examination of the existing literature on the relationship between vitamin D and type 2 diabetes. The analysis had a total of 7974 writers. Anastassios G. Pittas has authored 22 publications and has an h-index of 42, making him the most productive author, as indicated in **Table 5**. Nasser Al-Daghri has authored 16 articles and has an h-index of 51. On the other hand, Jorde, Rolf has published 12 articles and has an h-index of 63.

**Table 5:** Top 10 most published authors.

Rank	Author	TLS	TP	TC	H-index	Institution
1	Pittas, Anastassios G.	64	22	2048	42	Tufts Medical Center
2	Nasser Al-Daghri	38	16	466	51	King Saud University
3	Jorde, Rolf	28	12	924	63	Tufts Medical Center
4	Tirang R. Neyestani	44	12	663	25	Shahid Beheshti University Medical Sciences
5	Dawson-Hughes, Bess	38	10	1108	109	Tufts Medical Center
6	Li, Wenjie	42	10	117	22	Guilin University of Electronic Technology
7	Manson, JoAnn E.	13	10	929	139	Harvard University
8	Wang, Chongjian	40	10	124	3	Zhengzhou University
9	Bowden, Donald W.	69	9	237	71	Wake Forest University
10	John Jeffrey Carr	69	9	237	71	Vanderbilt University School of Medicine

**Figure 6:**
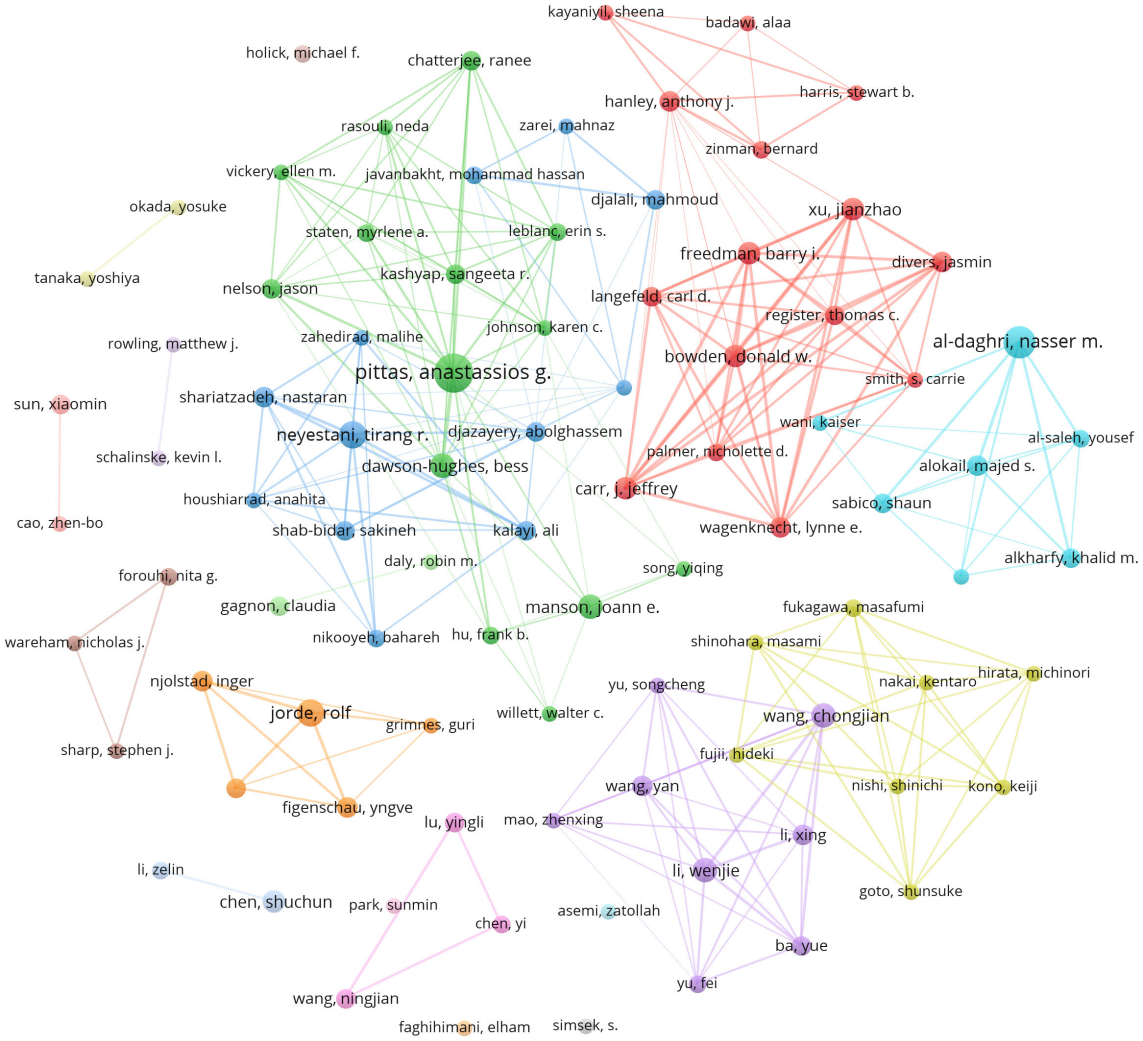
Network clustering of author co-authorship analysis.

Among the 7974 authors, only a mere 90 individuals managed to produce a minimum of 5 publications. We conducted a network analysis on the 90 writers, examining their co-authorship and temporal overlap. Circle size was used to represent the number of publications, while color was used to show clustering. The cluster analysis results indicate that collaborations predominantly occur inside countries, while direct cooperation between different countries is very infrequent. This indicates the necessity to bolster collaboration among worldwide research teams and augment the global interchange of pertinent research (**Figure 6**). Temporal co-occurrence network analysis revealed that researchers in China conducted relevant research later (**Supplementary Attachment 1**).

### 3.7 Frequency and clustering analysis of keywords

**Figure 7:**
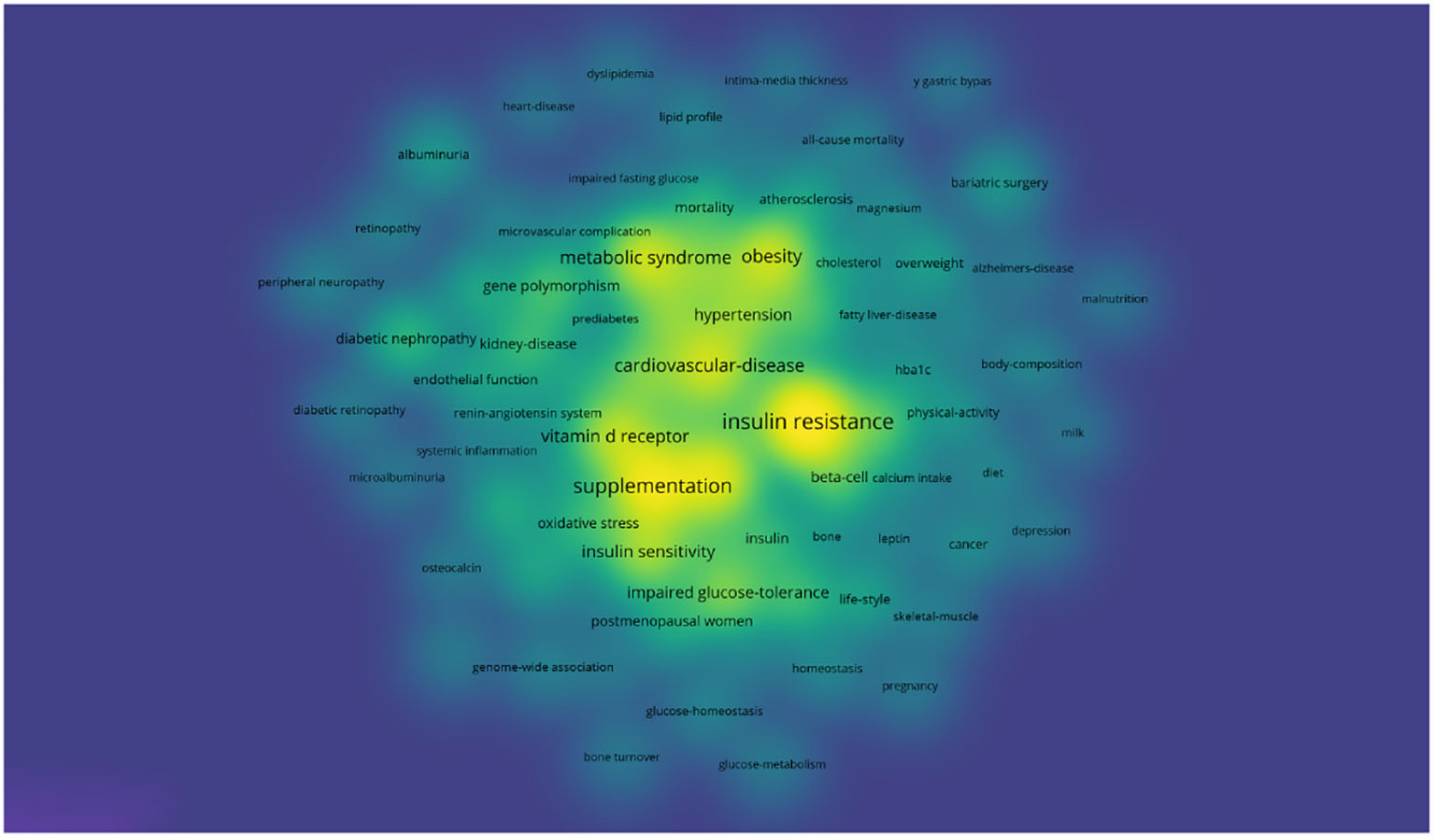
Density map of keywords.

Keyword co-occurrence analysis is a commonly used bibliometric method that reveals the relationships between research themes, disciplinary fields, or concepts by analyzing the co-occurrence of keywords in the research literature (23). In this study, keywords with the same meaning were merged, and ineffective keywords were eliminated, ultimately identifying 86 keywords that met the criterion of appearing at least ten times. By generating a density map, the distribution of popular themes across the field can be easily observed. As shown in **Figure 7**, the most popular elements are “cardiovascular disease,” “metabolic syndrome,” “insulin resistance,” “vitamin D receptor,” and “supplementation.” These elements are prominently displayed on the density map.

**Figure 8:**
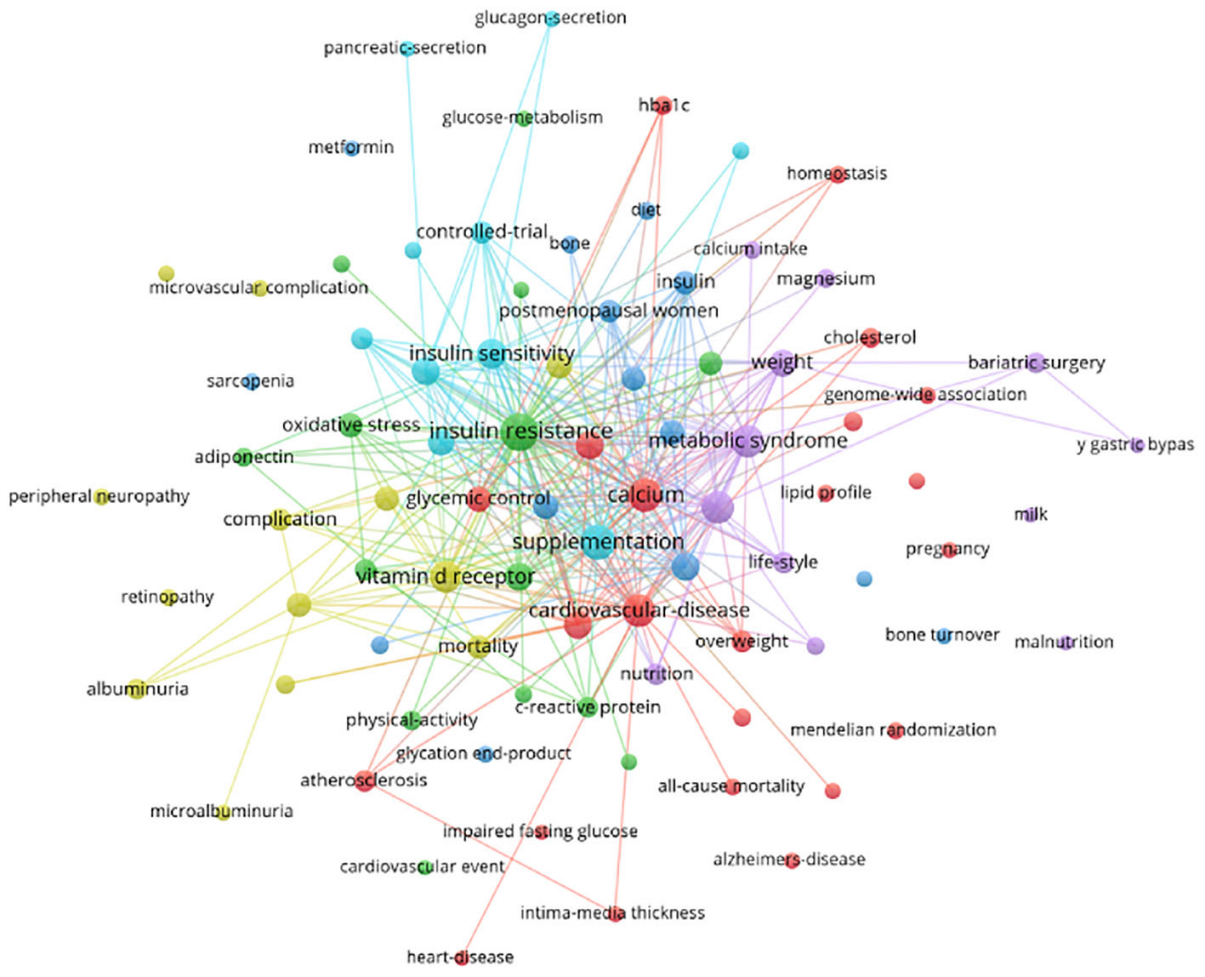
Co-occurrence network of keywords.

Subsequently, we conducted a clustering analysis using the selected keywords. Six clusters composed of 89 high-frequency keywords represent the six main research areas of the theme (**Figure 8**). Cluster 1 is the largest, marked with a red circle, primarily focusing on cardiovascular disease and lipid profile, containing a total of 23 keywords. Cluster 2 includes 14 keywords, represented by a green circle, mainly involving inflammation. Cluster 3, denoted by a dark blue circle, emphasizes body composition and bone metabolism. The yellow cluster mainly studies diabetic complications. The blue cluster primarily relates to diet and lifestyle. Cluster 6, in light blue, mainly focuses on mechanisms.

**Figure 9:**
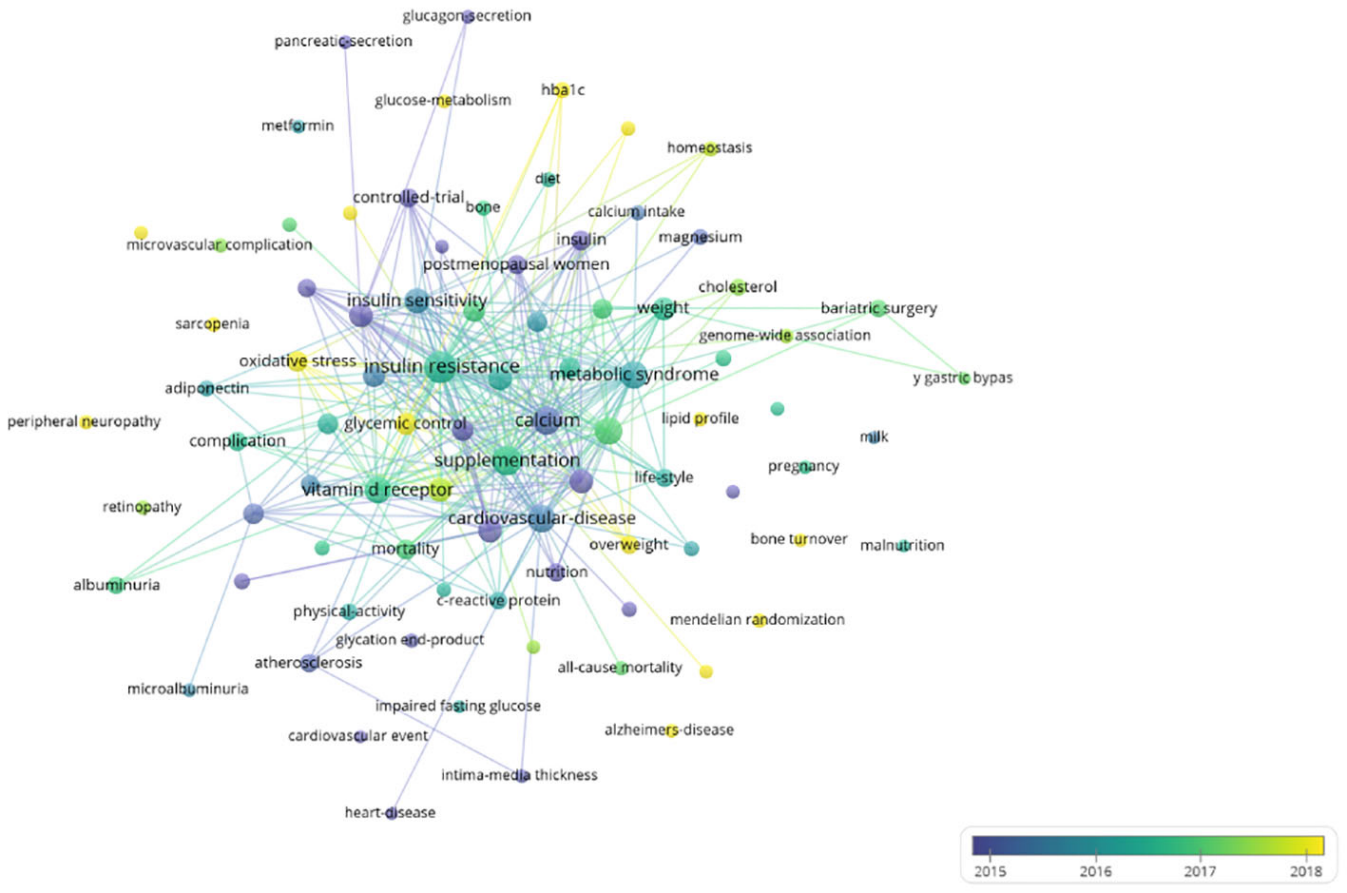
Time-overlapping network of keywords.

**Figure 10:**
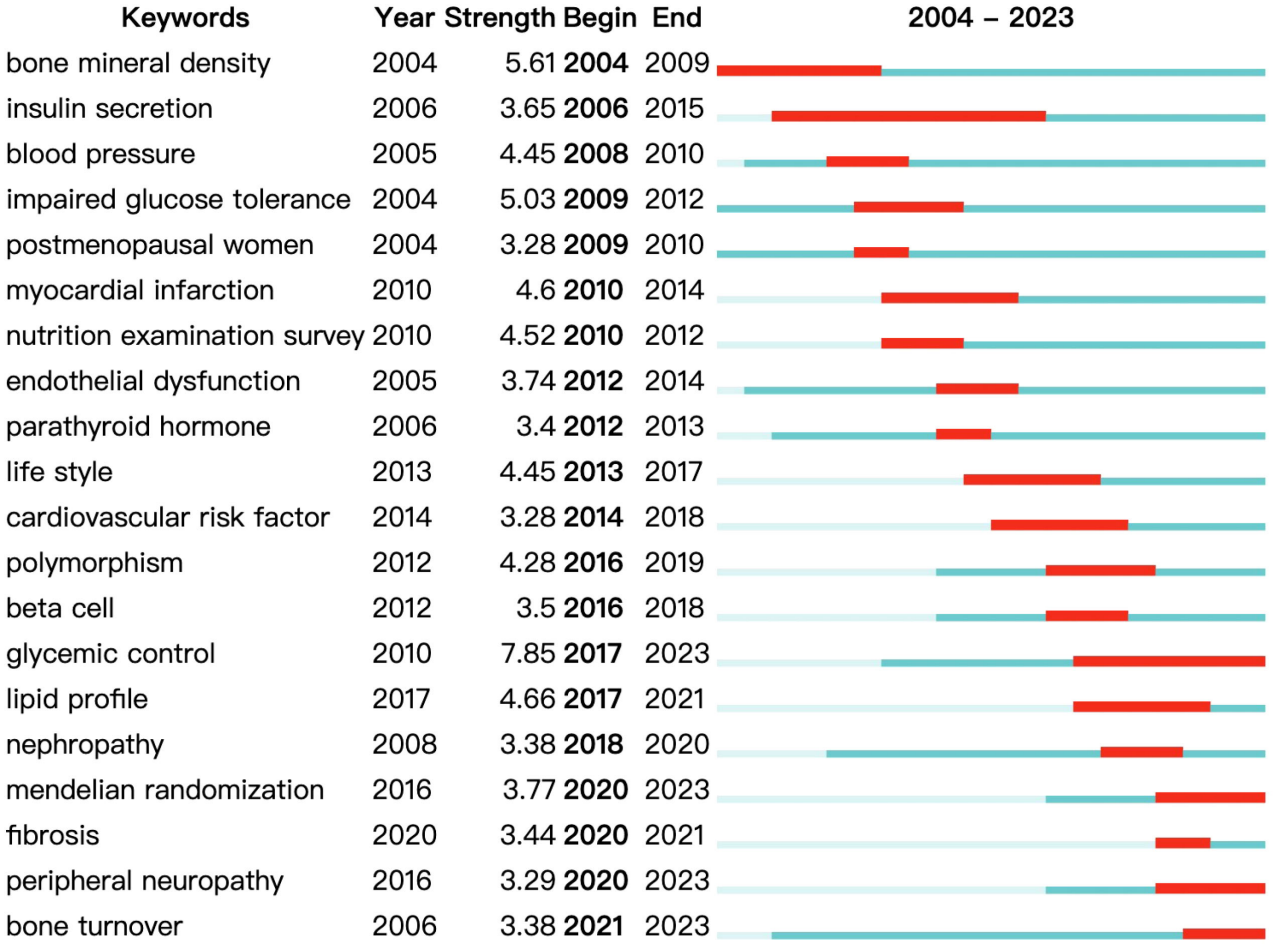
Top 20 keywords with the strongest citation bursts.

Finally, we present a graphical representation of the keyword network over time, as shown in **Figure 9**. Yellow indicates the most recent keywords, while purple represents the earlier ones. Early research focused on keywords such as “cardiovascular disease,” “glucagon secretion,” and “pancreatic secretion.” More recent studies concentrate on themes like “oxidative stress,” “peripheral neuropathy,” and “Alzheimer’s disease.” The keyword burst detection has shown that “bone turnover” and “peripheral neuropathy” are keywords that have continued to burst up to the year 2023 (**Figure 10**).

## 4 Discussion

This study utilizes bibliometric methods to provide a comprehensive evaluation of academic achievements related to vitamin D and type 2 diabetes research over the past two decades. We have identified publication trends, regional distributions, and collaborations among countries and institutions in articles related to this topic, and further evaluated highly cited papers. Additionally, our groundbreaking findings reveal that research on bone turnover and peripheral neuropathy might be promising directions for future investigations. These findings provide additional insights for research policymakers.

Drawing upon previous research and focusing on the highly cited literature identified in this study, we conducted a comprehensive literature review of the relationship between vitamin D and type 2 diabetes. The number of citations an article receives is an important indicator of the impact of academic achievements, reflecting to some extent the academic value and influence of the article, a significance that cannot be overlooked (24). In terms of clinical research, as early as 2006, a large prospective study indicated that women with higher daily intakes of vitamin D had a significantly reduced risk of developing diabetes (25). In 2013, a meta-analysis of prospective studies presented similar conclusions, finding a significant inverse relationship between 25(OH)D levels and the incidence of type 2 diabetes. This observed inverse correlation was not affected by gender, sample size of the study, duration of follow-up, criteria for diabetes diagnosis, and methods of measuring 25(OH)D (26). Similarly, an exposure-wide umbrella review of meta-analyses in 2018 indicated an inverse relationship between blood levels of vitamin D and the risk of type 2 diabetes (27). However, a multicenter, randomized, placebo-controlled trial in 2019 showed that daily supplementation with 4000 IU of vitamin D3 did not lead to a significant reduction in diabetes risk (28). In terms of the underlying mechanisms, a significant amount of research has been conducted by scientists. A study (29) in 2004 demonstrated that the concentration of 25(OH)D is positively correlated with insulin sensitivity and negatively correlated with β-cell function in cases of vitamin D deficiency. Subjects with vitamin D deficiency were found to be at a higher risk of insulin resistance and metabolic syndrome. A 2008 study showed that a single large dose of oral vitamin D2 could improve endothelial function in patients with type 2 diabetes and vitamin D deficiency. Further studies have indicated a negative correlation between baseline serum 25(OH)D levels and future blood glucose and insulin resistance (30). A clinical trial in 2011 revealed that vitamin D supplementation could improve the disposition index and insulin secretion, associating vitamin D supplementation with improved pancreatic β-cell function in adults at high risk for type 2 diabetes, and it showed a trend toward reducing the rise in HbA1c (31). Vitamin D supplementation also has certain clinical application value in preventing diabetes complications. Adding paricalcitol to RAAS inhibitors can safely reduce residual albuminuria in patients with diabetic nephropathy and may represent a new method to reduce the residual renal risk in diabetic patients (32). Additionally, vitamin D is related to peripheral nerve conduction; patients with vitamin D deficiency often experience more neuropathic deficits associated with diabetic peripheral neuropathy. Vitamin D deficiency is also considered a risk factor for diabetic retinopathy (33). Clinical studies have demonstrated that vitamin D supplementation is effective in treating diabetic retinopathy (34). However, additional large-scale studies are needed to determine the optimal timing and duration of vitamin D intervention in type 2 diabetes and its complications.

Burst detection is an important feature in CiteSpace, primarily used to identify and analyze the sudden growth of keywords or topics within a certain period, thereby revealing emerging trends and hotspots in research fields (35). “Bone turnover” and “peripheral neuropathy” are keywords that have continued to burst up to the year 2023. Studies have shown that the concentration of 25(OH)D is negatively correlated with insulin resistance and bone turnover. Insulin resistance increases with the decrease in 25(OH)D concentration, which can enhance bone turnover and increase the risk of osteoporosis in non-osteoporotic type 2 diabetes patients (36). Furthermore, research has found that vitamin D may play a direct or indirect significant role in the positive correlation between bone metabolism and basal metabolism. This relationship is more evident in patients with increased serum 25(OH)D levels and those receiving Alfacalcidol supplementation. This suggests that improving vitamin D levels and Alfacalcidol supplementation may help improve bone metabolism and basal metabolism in postmenopausal women with type 2 diabetes (37). Vitamin D is related to the conduction capability of peripheral nerves and may have a neuro-selective and threshold-selective relationship with the incidence and severity of diabetic peripheral neuropathy in type 2 diabetes patients. In summary, research related to diabetes and bone metabolism, and vitamin D and diabetic neuropathy, are current research hotspots and future research trends (37).

To our knowledge, this is the first bibliometric analysis in this field. Existing publications focus mainly on isolated studies, such as individual mechanisms. Our study offers a comprehensive analysis of global literature on Vitamin D and type 2 diabetes, highlighting future research hotspots and trends to better assist researchers. However, this study also has some limitations. The quantitative indicators commonly used in bibliometrics, although capable of reflecting the impact of scientific achievements to some extent, may be influenced by various factors such as disciplinary fields, the age of the literature, and the popularity of research topics, and do not equate directly to research quality or impact. Furthermore, this study is primarily based on published scientific literature, which may overlook important information contained in unpublished research, academic conference reports, and other informal publications.

## Data Availability

Publicly available datasets were analyzed in this study. This data can be found here: https://webofscience.clarivate.cn/wos/alldb/basic-search.
