# Supplementary material for: Research trends in the relationship between vitamin D and type 2 diabetes mellitus: a 20-year bibliometric and visualization analysis
Source: Front Endocrinol (Lausanne). 2024 Aug 13;15:1421953. doi: 10.3389/fendo.2024.1421953 (PMC11347281; doi:10.3389/fendo.2024.1421953)

**A** The dual-map overlay of journals publishing studies on vitamin D and type 2 Diabetes.


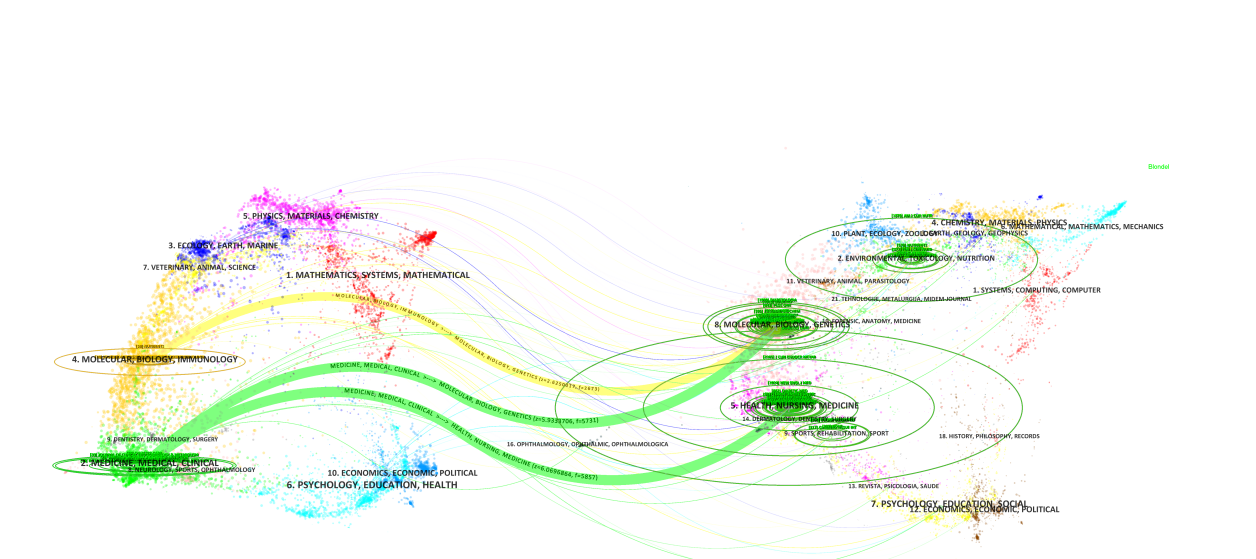


**B** Time-overlapping network of country co-authorship analysis.


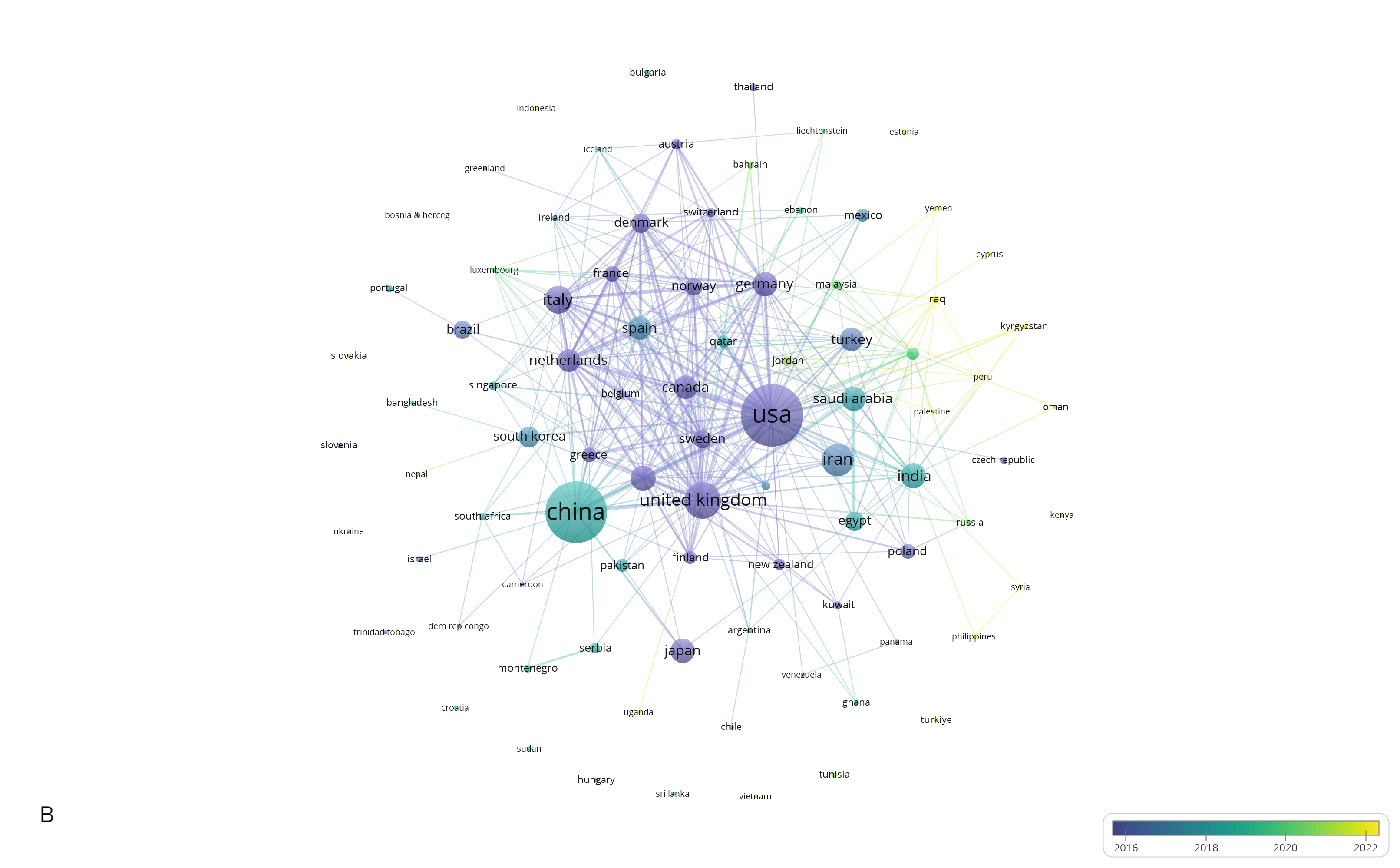


**C** Time-overlapping network of institution co-authorship analysis.


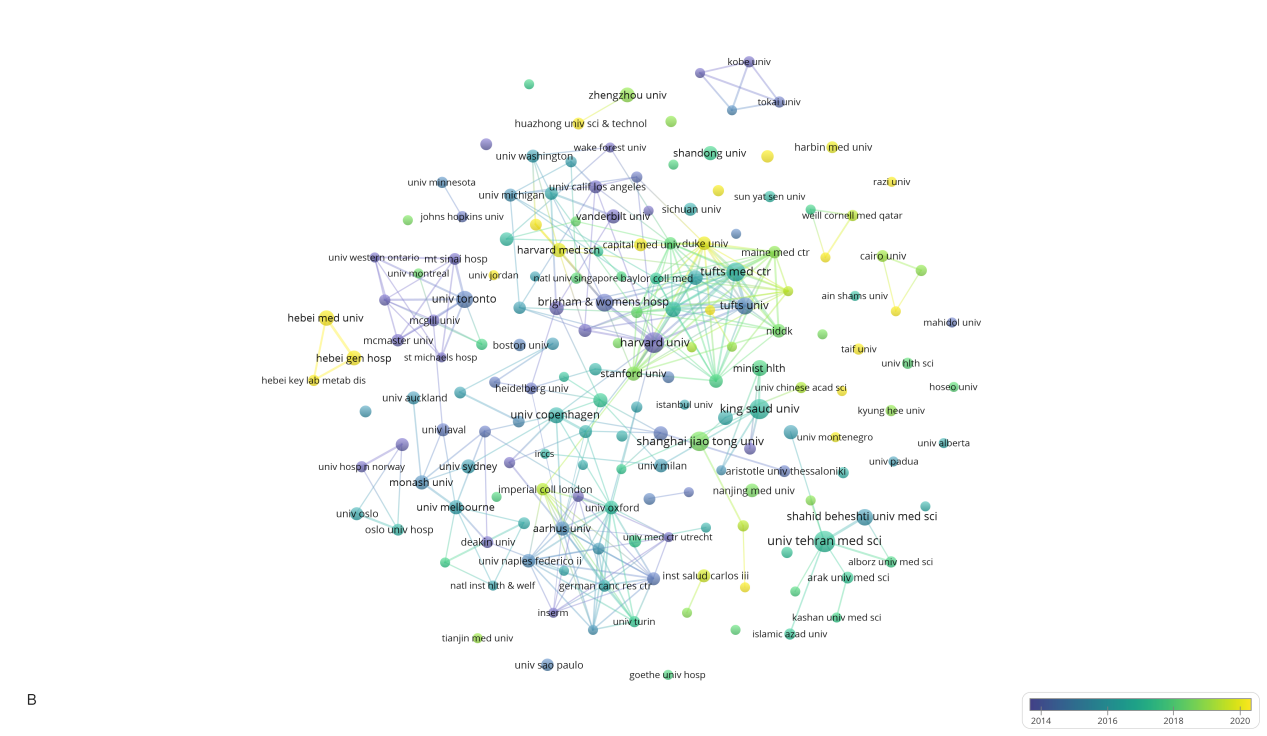


**D** Time-overlapping network of author co-authorship analysis.


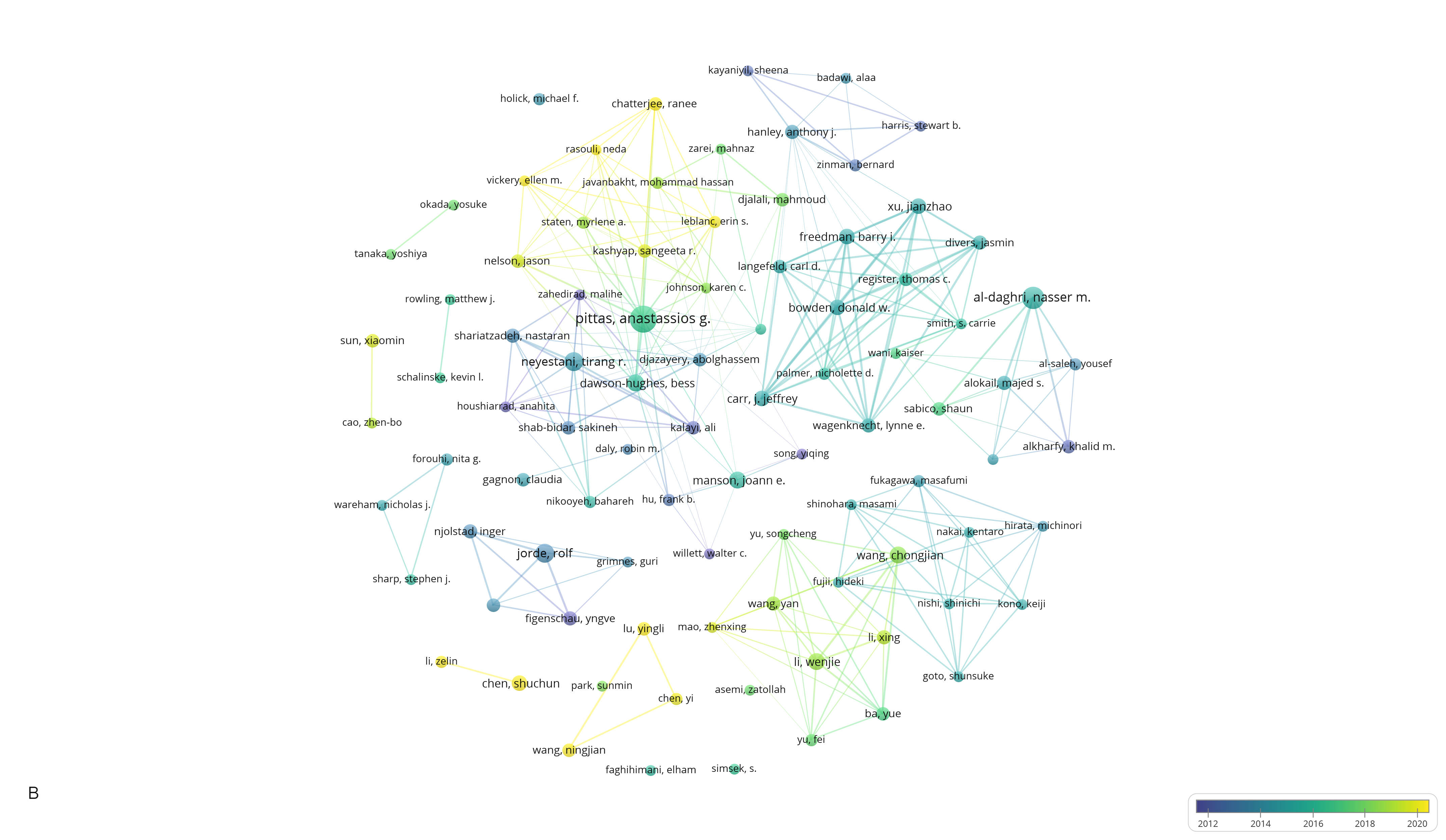

Supplement: Supplementary file 1 [file DataSheet1.docx]
